# A cluster randomized trial comparing deltamethrin and bendiocarb as insecticides for indoor residual spraying to control malaria on Bioko Island, Equatorial Guinea

**DOI:** 10.1186/s12936-016-1433-0

**Published:** 2016-07-22

**Authors:** John Bradley, Dianna Hergott, Guillermo Garcia, Jo Lines, Jackie Cook, Michel A. Slotman, Wonder Philip Phiri, Christopher Schwabe, Immo Kleinschmidt

**Affiliations:** MRC Tropical Epidemiology Group, London School of Hygiene and Tropical Medicine, London, UK; Medical Care Development International, Silver Spring, MD USA; Department of Disease Control, London School of Hygiene and Tropical Medicine, London, UK; Texas A&M University, College Station, TX USA; Department of Pathology, School of Health Sciences, University of Witwatersrand, Johannesburg, South Africa

## Abstract

**Background:**

Indoor residual spraying (IRS) has been used on Bioko for malaria control since 2004. In 2013 the insecticide was changed from bendiocarb to deltamethrin. Shortly after this change, there was a marked increase in malaria prevalence on the island. This trial was carried out to compare the effectiveness of bendiocarb and deltamethrin for use in IRS on Bioko.

**Methods:**

Twenty-four clusters of houses were randomized to receive IRS with either bendiocarb or deltamethrin. Approximately 3 months after the intervention, the prevalence of malaria and levels of haemoglobin were measured in children aged 2–14 years in each cluster.

**Results:**

Prevalence of malaria in 2–14 year olds was lower in the bendiocarb arm (16.8, 95 % CI 11.1–24.7, N = 1374) than in the deltamethrin arm (23.2, 95 % CI 16.0–32.3, N = 1330) but this difference was not significant (*p* = 0.390), even after adjusting for covariates (*p* = 0.119). Mean haemoglobin in children was marginally higher in the bendiocarb clusters (11.6 g/dl, 95 % CI 11.5–11.8, N = 1326) than in the deltamethrin clusters (11.5 g/dl, 95 % CI 11.3–11.7, N = 1329). This difference was borderline significant after adjusting for covariates (*p* *=* 0.049).

**Conclusions:**

The results are suggestive of bendiocarb being more effective at preventing malaria on Bioko although evidence for this was weak. The results are likely due to the fact that local vectors remain fully susceptible to bendiocarb whereas subsequent tests have shown resistance to deltamethrin.

## Background

Indoor residual spraying (IRS)—spraying the interior walls of houses with insecticide—is a highly effective form of vector control with a long history in malaria prevention [1]. It was the mainstay of the Global Malaria Eradication Program launched by the World Health Organization (WHO) in 1955, responsible for eliminating malaria from large parts of the world [2]. IRS is used extensively: in 2014, 50 million people in sub-Saharan Africa were protected by IRS, representing 6 % of the population at risk in the region [3].

Bioko Island has had a historically high malaria burden. To combat this, the Bioko Island Malaria Control Project (BIMCP) was launched in 2004 [4]. The Project has had considerable success in reducing malaria prevalence and child mortality [5, 6]; and much of this success has been attributed IRS [7].

The first IRS round on the island took place in 2004 using the insecticide deltamethrin. Deltamethrin IRS was discontinued after one spray round as a precaution because the *kdr* mutation was detected at high frequency in *Anopheles gambiae* s.s. [4]. IRS with the insecticide bendiocarb was introduced in 2005 to maintain the effectiveness of IRS and reduce selection pressure for resistance to pyrethroids [4]. Bendiocarb was sprayed biannually on Bioko until 2013, when policy changed to one spray round a year with a long lasting formulation of deltamethrin. The decision to change was taken for several reasons: (1) detailed analysis of pyrethroid resistance in *An. gambiae* on Bioko in 2011 showed no evidence that P450-based metabolic resistance mechanisms were present [8]; (2) re-analysis of data from 2004 indicated that deltamethrin in fact was effective at controlling local vectors [8]; (3) rotation of insecticides is recommended by the WHO to manage insecticide resistance [9]; (4) bendiocarb has a short residual life and had been shown to leave the population unprotected for parts of the year [10, 11] and (5) the new long acting formulation of deltamethrin required only one spray round a year, reducing the cost.

After the first spray round with the new deltamethrin formulation in 2013, malaria infection prevalence in 2–14 year old children, measured annually in an island wide cross-sectional malaria indicator survey, rose from 14 % in 2012 to 28 % in the following year. This sharp rise in prevalence prompted the BIMCP to carry out the trial reported here to compare the effectiveness of deltamethrin and bendiocarb as an IRS insecticide on Bioko Island.

## Methods

### Study setting

Bioko Island is part of Equatorial Guinea. It has an area of approximately 2000 km^2^ and lies 32 kms off the coast of Cameroon in Central Africa. The population is approximately 250,000, with the majority of people living in Malabo, the island’s major city. A malaria indicator survey in 2004, before the start of island wide IRS, found a parasite prevalence of 46 % in children aged from 2 to 14 years [6]. IRS has been the predominant form of vector control used by the BIMCP, but in addition there was a single mass distribution of long-lasting insecticidal nets (LLINs) in 2008, and continuous distribution of LLINs to pregnant women as part of antenatal care. High coverage of LLINs has not been maintained: only 13 % of 2–14 year old children reported sleeping under a LLIN in 2013 (BIMCP, unpublished observations).

The most important malaria vectors on Bioko are *An. gambiae* s.s. throughout the island, and *Anopheles melas* in coastal areas [12–14]. The BIMCP measures entomological indicators at ten sentinel sites throughout the island. This includes monitoring the susceptibility of local mosquitoes to insecticides used in malaria control [8] using the standard WHO test [15]. In 2014, 24-h mortality of local *An. gambiae* s.s was 100 % (N = 80) after exposure to bendiocarb and 79.6 % (N = 240) after exposure to deltamethrin (BIMCP unpublished observations). Deltamethrin continued to be used by BIMCP as part of a planned long-term insecticide rotational scheme and because tests had shown metabolic resistance not to be present on the island [8].

### Trial design

**Fig. 1:**
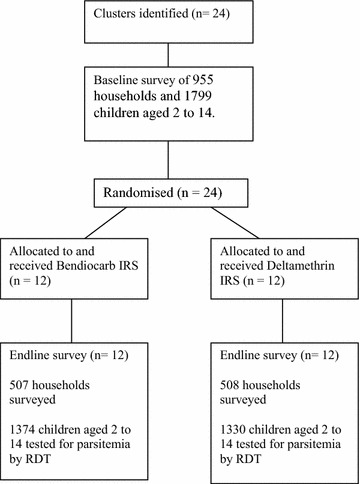
Overview of trial

**Fig. 2:**
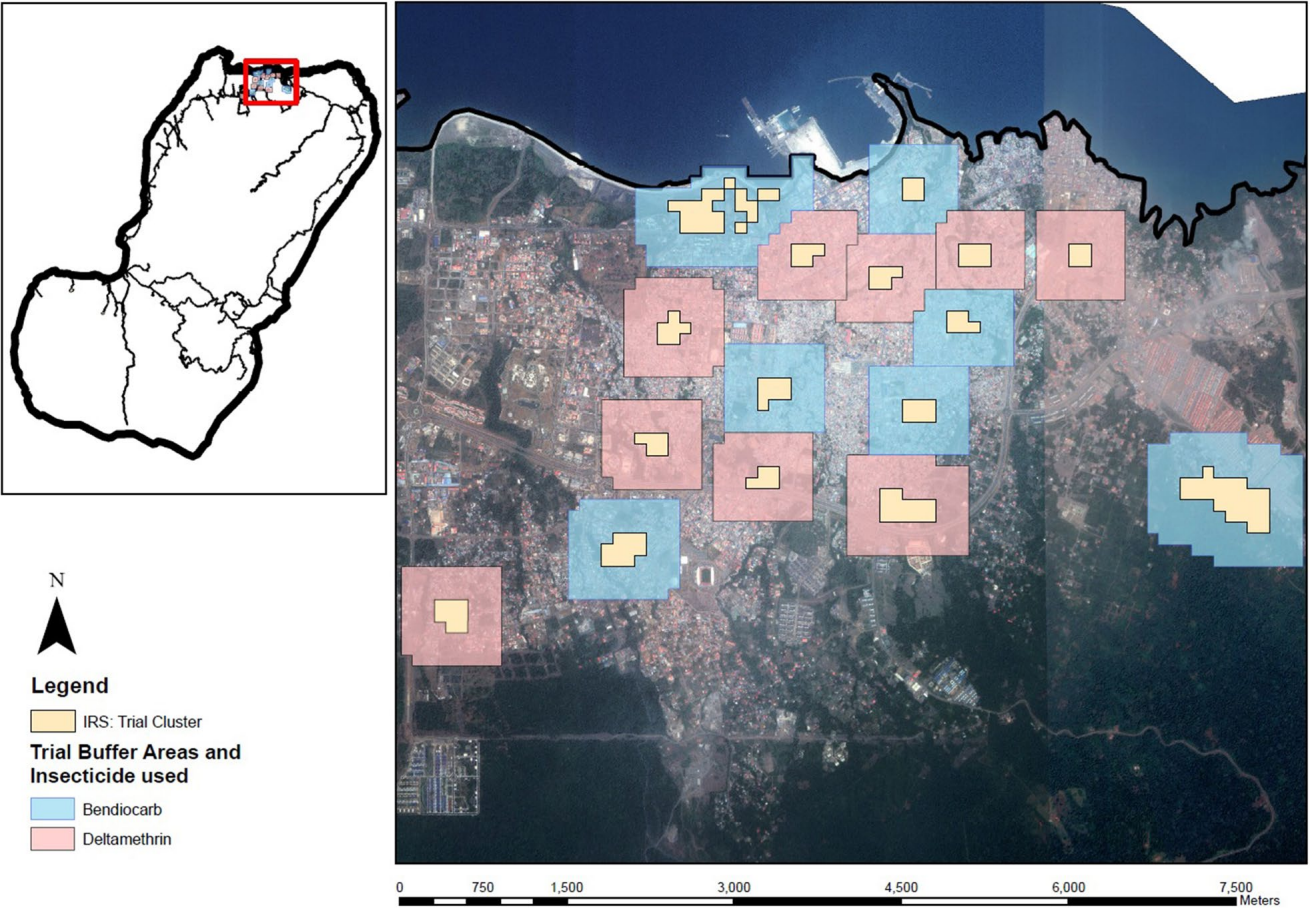
The trial clusters in Central Malabo. The *shaded area* around the cluster represents the buffer zone. *Pink* clusters were sprayed with deltamethrin and *blue* clusters with bendiocarb

Clusters of households were randomized to two trial arms, one receiving bendiocarb IRS, the other receiving deltamethrin IRS (Fig. 1). These clusters were formed using the BIMCP mapping system which has divided the island up into 100 m^2^ sectors to facilitate routine IRS. Clusters constituted groups of adjacent sectors to make up at a minimum of 250 households whilst ensuring a distance of at least 300 m between the borders of adjacent clusters (Fig. 2). Neighbourhoods known to have a high rate of refusing IRS in the past were not eligible for participation in the trial. Eighteen clusters in urban Malabo were selected, along with six clusters in rural locations.

The primary comparison between the arms was the prevalence of *Plasmodium falciparum* malaria in children aged from 2 to 14 years. Two secondary comparisons were made: mean haemoglobin on the same set of children; and the proportion of households willing to have their house sprayed with the insecticide in future spray rounds.

Sample size calculations were made assuming a mean prevalence of *P. falciparum* of 28 % in the deltamethrin arm and 14 % in the bendiocarb arm. These prevalences are the estimated island wide prevalences in 2013 and 2012 when deltamethrin and bendiocarb were used for IRS respectively. The coefficient of variation between clusters was assumed to be 0.25—this is suggested in the methodological literature as a reasonable value to assume in the absence of data [16]. Under these assumptions, with 12 clusters in each arm and 120 children recruited for the survey in each cluster, the trial had over 90 % power to detect a difference in prevalence between the arms at the 5 % significance level.

Restricted randomization was used to achieve balance between the arms on variables expected to be predictive of malaria outcomes [17]. Data on these variables was collected in a cross sectional baseline survey of 40 randomly selected households per cluster in March 2014. The restrictions were: each arm had to have three of the six rural clusters, and the mean of the following variables could not differ by more than 10 % between trial arms: the baseline prevalence of malaria in children aged from 2 to 14 years measured by rapid diagnostic test (RDT) (Carestart, Access-Bio Inc., Monmouth, USA); the proportion of households with at least one LLIN per two people; the proportion of households wanting IRS; and the proportion of houses that received IRS in the last spray round.

Two hundred thousand allocations were generated at random, 6304 of which satisfied the imposed restrictions. After checking that these 6304 randomizations allowed independence of allocation between pairs of clusters, one of the eligible allocations was chosen at random.

### Interventions

The intervention took place in March and April 2014. All households in the trial were offered IRS according to the study arm they had been allocated to. The two insecticides were bendiocarb (Ficam™, Bayer) in the insecticide class of carbamates, and a long lasting formulation of deltamethrin (K-Othrine™, Bayer) in the class of pyrethroids. Both insecticides are approved for public health use by the WHO Pesticide Evaluation Scheme (WHOPES) [18, 19]. Spraying was done at the WHOPES recommended dose (0.02–0.025 and 0.1–0.4 g of active ingredient per square metre for deltamethrin and bendiocarb, respectively). Bendiocarb has a residual life of 2–6 months and deltamethrin has a residual life of 6 months [18]. Spray teams aimed to spray at least 85 % of households in each cluster. If less than 85 % of households were sprayed on the first visit, the spray teams made further visits to the cluster until either 85 % coverage had been reached or the team had returned three times. It was explained to households that they had the right to refuse IRS.

Each cluster was surrounded by a buffer zone of 300 m in which houses were sprayed with the same insecticide as the houses in the cluster. In cases where the distance between two clusters was less than 600 m, the buffer zone extended to half way between the clusters (Fig. 2).

### Evaluation

The effect of the interventions was evaluated by a cross sectional household survey in June and July 2014, 11–13 weeks after spraying. The evaluation was timed to precede expiry of the residual life of both insecticides. Databases of households used by the BIMCP in the routine IRS programme were used to obtain a random sample of 60 households per cluster. Surveyors visited each selected household and, if any children aged 2–14 years were present, sought written informed consent from the caregiver/householder to take part in the trial. At each participating household three children aged from 2–14 years were randomly selected (or all children if there were three or fewer present) and were tested for parasitaemia by RDT (Carestart, Access-Bio Inc., Monmouth, USA) and had their haemoglobin measured (HemoCue, Ängelholm, Sweden). An adult was asked whether their house was sprayed, if they liked the insecticide they received and whether they would accept it again in the next spray round. They were also asked about ownership and use of bed nets. The endline survey took place only in the core area of each cluster, not in the buffer zone.

### Statistical analysis

Parasitaemia, spray coverage and acceptability of IRS were analysed as binary outcomes using logistic regression. Generalized estimating equations with exchangeable working correlation matrix and robust standard errors were used to account for intra cluster correlation of responses. Haemoglobin level was analysed as a continuous outcome using linear regression with a cluster level random intercept to account for intra cluster correlation. All analyses were carried out as intention to treat, so that all clusters were included regardless of the level of coverage received. Analyses adjusting for baseline malaria prevalence, sleeping under a bed net the night before the survey, reported IRS coverage, age, and rural vs urban location were carried out as secondary analysis. All analyses were done using Stata version 13 [20].

### Ethics approval

Ethics approval for the trial was granted by the Equatorial Guinea Ministry of Health and Social Welfare and the ethics committee of the London School of Hygiene and Tropical Medicine (approval number 8048). Written informed consent was obtained from all participants. In both the baseline and endline surveys, children testing positive for malaria were given standard first line anti-malarial treatment according to national policy (Artemisinin plus amodiaquine) from a trial nurse. Children with anaemia were referred to a local health facility for further treatment.

## Results

### Baseline

In the baseline survey, 955 households were surveyed and 1799 children were tested for malaria. Baseline characteristics were similar between the two trial arms (Table 1). Baseline prevalence in the bendiocarb arm was 17.5 % (95 % CI 12.3–24.3) compared with 18.4 % (95 % CI 12.7–25.9) in the deltamethrin arm. The range of baseline prevalences by cluster was 2.0–41.6 %. There were similar proportions of houses receiving IRS in the bendiocarb and deltamethrin arms (85.0 vs 83.5 % respectively); willing to accept IRS (89.2 vs 91.4 %); owning as least one net (64.1 vs 66.8 %); and having at least one net for every two persons (26.7 vs 25.0 %).

**Table 1:** Baseline variables for the 24 trial clusters

	Bendiocarb arm	Deltamethrin arm	Total
Malaria prevalence in children aged 2–14 years, measured by RDT			
% [95 % CI] (N)	17.5 [12.3–24.3] (845)	18.4 [12.7–25.9] (954)	18.0 [14.0–22.8] (1799)
Range, %	5.3–38.9	2.0–41.6	2.0–41.6
Houses reporting receiving IRS in the last year			
% [95 % CI] (N)	85.0 [79.9–89.0] (340)	83.5 [75.4–89.2] (387)	84.2 [79.6–87.9] (727)
Range, %	64.7–100	63.7–100	63.7–100
Households willing to accept IRS			
% [95 % CI] (N)	89.2 [86.1–91.2] (406)	91.4 [86.7–94.6] (466)	90.4 [87.6–92.5] (872)
Range, %	78.6–96.7	72.4–100	72.4–100
Households owning at least one net			
% [95 % CI] (N)	64.1 [50.0–76.2] (379)	66.8 [57.3–75.1] (425)	65.6 [57.4–72.9] (804)
Range, %	24.2–90.5	50.0–96.7	24.2–96.7
Households with at least one net per 2 people			
% [95 % CI] (N)	26.7 [18.5–36.9] (378)	25.0 [16.9–35.2] (424)	25.8 [19.9–32.7] (802)
Range, %	9.5–53.4	6.3–66.7	6.3–66.7

### Parasitaemia and haemoglobin post-intervention

In the endline survey, 1015 households were visited; 2704 children were tested for parasitaemia; and 2655 haemoglobin measurements were recorded (Table 1). Prevalence of malaria in 2–14 year olds was lower in the bendiocarb arm (16.8, 95 % CI 11.1–24.7, N = 1374) than in the deltamethrin arm (23.2, 95 % CI 16.0–32.3, N = 1330) but this difference was not significant (*p* = 0.390), even after adjusting for covariates (*p* = 0.119) (Table 2). There was substantial variability in endline malaria prevalence: the range in the bendiocarb clusters and deltamethrin clusters was 1.6–40.9, and 1.3–50.8 % respectively. Mean haemoglobin was marginally higher in the bendiocarb clusters (11.6 g/dl, 95 % CI 11.5–11.8, N = 1326) than in the deltamethrin clusters (11.5 g/dl, 95 % CI 11.3–11.7, N = 1329), with very weak evidence for a difference (*p* = 0.302). After adjusting for covariates, the difference in haemoglobin between study arms was borderline significant (*p* = 0.049).

**Table 2:** Outcome measures at endline

	Bendiocarb arm	Deltamethrin arm
Malaria prevalence in children aged 2–14 years, measured by RDT		
% [95 % CI] (N)	16.8 [11.1–24.7] (1374)	23.2 [16.0–32.3] (1330)
Range, %	1.6–40.9	1.3–50.8
OR [95 % CI]	–	1.33 [0.70–2.54] p = 0.390
Adjusted^a^ OR [95 % CI]	–	1.50 [0.86–2.64] p = 0.154
Haemoglobin, g/dl		
Mean [95 % CI] (N)	11.63 [11.46–11.80] (1326)	11.50 [11.33–11.67] (1329)
Range, %	11.08–11.98	10.80–12.07
Difference [95 % CI]	–	0.13 [0.11–0.37] p = 0.302
Adjusted^a^ difference [95 % CI]	–	0.13 [0.00–0.27] p = 0.049

^a^Adjusted for baseline malaria prevalence, sleeping under a bet the night before the survey, reported IRS coverage, age and rural vs urban location

### Coverage

Mean reported IRS coverage was 73.7 % (95 % CI 67.1–79.4, N = 495) in the bendiocarb arm and 76.5 % (95 % CI 71.6–80.9, N = 490) in the deltamethrin arm. Reported coverage ranged from 50.0 to 84.9 % in the bendiocarb clusters and from 64.7 to 100.0 % in the deltamethrin clusters. The difference in coverage between the arms was not significant (p = 0.368).

### Acceptability

In response to the question *Did you like the insecticide that was used to spray your house*? 85.1 % (95 % CI 80.4–88.8, N = 295) of households that received bendiocarb said yes, compared to 85.3 % (95 % CI 79.5–89.7, N = 292) of households that received deltamethrin (p = 0.959).

In response to the question *If the same insecticide is used in the next round, would you accept to have your house sprayed*? 92.8 % (95 % CI 89.3–95.2, N = 304) of households that received bendiocarb said yes, compared to 92.5 % (95 % CI 88.8–95.1, N = 310) of households that received deltamethrin (p = 0.933).

### Use of bed nets

In the bendiocarb arm, 30.1 % (95 % CI 20.8–41.3) of 2–14 year old children slept under aLLIN the night before the survey, compared to 31.7 % (95 % CI 18.9–48.1) in the deltamethrin arm (p = 0.368).

## Discussion

The aim of this trial was to compare the effectiveness of deltamethrin and bendiocarb as an IRS insecticide on Bioko Island—and in particular to determine if the rise in malaria prevalence on Bioko in 2013 could be attributed to changing from bendiocarb to deltamethrin IRS. The results are inconclusive. The prevalence of malaria in children was markedly lower in bendiocarb clusters than in deltamethrin clusters-but this was not statistically significant so the difference could be due to chance. The evidence for a difference in haemoglobin was stronger-but only after adjusting for confounders, and the difference was too small to be clinically significant.

There are several aspects of the trial design and implementation that could have contributed to a non-significant result. In a cluster randomized trial of an infectious disease it is important that clusters be big enough to capture *mass effects* of an intervention: in this trial it would be the benefit derived from surrounding houses receiving IRS, whether or not your own house was sprayed. It is also important that clusters are sufficiently far apart to avoid *spillover effects*: in this case residents of a cluster benefiting from vector suppression in a neighbouring cluster [21]. In an ideal world clusters should be as large as possible and as far away from each other as possible, but in the real world this must be balanced against logistic, financial and geographical constraints. In this trial, clusters comprised at least 250 houses and the border of each cluster was at least 300 m from the border of another cluster. It is not known how big or far apart clusters have to be in a trial of vector control for malaria prevention; but there is some evidence that spill-over effects of vector control interventions may be restricted to approximately 300 m [22]. It is possible that if the trial had used bigger clusters which were further apart, then a statistically significant result may have been obtained. In addition, households which were not in a cluster area were sprayed with the long-lasting deltamethrin product during the spray round, possibly diluting the effect of bendiocarb. Logistical constraints prevented a trial with larger separation between clusters from being implemented.

The power of the trial is affected by the amount of inter cluster variation in the primary outcome: the higher the variation, the lower the power. Since there were no data on variation of cluster level malaria prevalence, a coefficient of variation of 0.25 was assumed for sample size calculations; as suggested in the methodological literature [16]. However, baseline cluster level malaria prevalence in 2–14 year olds ranged from 2.0 to 41.6 %, and the estimated coefficient of variation was 0.59. If the sample size calculation is repeated with a coefficient of variation equal to 0.59 rather than 0.25, then power is only 68 %. The large inter cluster variation could be a reason that the trial did not find a statistically significant result. In the light of the high variation in baseline prevalence, it may have been wise to consider matching or stratification as ways of increasing the trial power [16].

In this trial only 24 clusters were randomized and this may not have been enough to achieve balance on all confounding factors. Although efforts were made to minimize bias through restricted randomization and regression adjustments, there may be some residual bias due to unmeasured confounders. For example, house construction [23] and prevalence of travel to the mainland [24] have both been shown to be important risk factors for malaria infection on Bioko but were not measured at either baseline or endline.

The trial took place over a short period of time. IRS can have a rapid effect on malaria transmission [1], but it is possible that in this case 3 months was not long enough to have an effect on malaria infection prevalence, even if there was a difference in effectiveness between the two insecticides. It would have been instructive to have entomological endpoints such as mosquito parity or entomological inoculation rate as secondary outcome measures, because IRS may affect these measures over a shorter time period. Unfortunately, this was not possible due to logistical constraints. Conversely, since bendiocarb has been shown to wane in effectiveness 3 months after spraying [10], it is also possible that a *smaller* difference between the trial arms would have been detected had the trial taken place over a longer period.

The WHO recommends that IRS coverage be at least 80 % to be optimally effective [3] which has been confirmed by previous studies in Bioko [25]. Reported coverage in this trial was 73.7–76.5 % in the bendiocarb and deltamethrin arms, respectively, which could have reduced the effectiveness of IRS in both arms and lessened the difference between them.

The results of this study have to be interpreted in the particular context of Bioko rather than simply as the effectiveness of the two insecticides per se. Resistance of *Anopheles* mosquitoes to insecticides is widely seen as a threat to malaria control in Africa [26, 27]—and there have been persistent concerns of resistance of local vectors to deltamethrin. Over the course of the BIMCP, the insecticide used for IRS has been switched twice. In 2005, the IRS insecticide was changed to bendiocarb after the first round of deltamethrin spraying in 2004, because a large proportion of *An. gambiae s.s.* caught in window exit traps had the *kdr* mutation—indicative of pyrethroid resistance [4]. Bendiocarb was subsequently sprayed twice a year until 2012, during which time malaria prevalence declined substantially [23].

Although vectors were shown to remain susceptible to bendiocarb, and PCR analysis showed that the proportion of *An. gambiae* s.s. with the *kdr* mutation remained high-the BIMCP reverted to deltamethrin as its IRS insecticide in 2013. This switch was prompted by a number of factors: (1) reanalysis of samples and data from 2004 showed that post-spraying with deltamethrin, *An. gambiae* s.s. mosquitoes with the *kdr* mutation did not have a higher sporozoite rate than those without the mutation and that IRS with deltamethrin had significantly reduced the abundance of *An. gambiae* s.s. [8]; (2) *An. gambiae* s.s. collected in 2011 showed no evidence of metabolic resistance to deltamethrin [8]; and (3) annual IRS with long-lasting deltamethrin was considerably less costly than biannual IRS with bendiocarb. However, WHO bioassay testing after the 2013 change in insecticide showed a loss of susceptibility to deltamethrin in local vectors. In standard WHO bioassays, 100 % (N = 100) of *An. gambiae* s.s. mosquitoes died within 24 h of exposure to bendiocarb in 2014 and 2015. However, mortality to deltamethrin was only 79.6 % (N = 240) and 29 % (N = 100) in 2014 and 2015 respectively (BIMCP, unpublished observations). In 2015, in response to the low mortality rate to deltamethrin, the BIMCP carried out a further metabolic resistance study in the mosquito population in Bioko Island in collaboration with the Liverpool School of Tropical Medicine. The results showed an up-regulation of P450-metabolic resistance in the Bioko mosquito population, indicating the lower susceptibility of the mosquitoes to pyrethroid insecticides (Hemingway, personal communication).

The BIMCP has discontinued the use of deltamethrin for IRS and has reverted to two rounds of bendiocarb in 2016. Resistance to bendiocarb in the mosquito population in Bioko will be monitored, and a rotational insecticide scheme with products that have different modes of action, as suggested by the WHO [9], is planned. In 2014 and 2015 (after this trial), the BIMCP carried out an island-wide mass-distribution with LLINs. Another mass distribution is planned in 2018. Due to pyrethroid resistance, the BIMCP is now only distributing Permanet 3.0 nets (Vestergaard) which contains a synergist as well as deltamethrin, increasing its efficacy against pyrethroid resistant malaria vectors. Since distributions of LLINs will be more frequent than before, the BIMCP expects that LLIN coverage will remain high with these new efforts. Beginning in 2015, the BIMCP targeted just 30 % of all households on Bioko with IRS, selecting communities most at-risk of malaria to receive bendiocarb IRS.

## Conclusions

The results of this trial are inconclusive by themselves, but they suggest that bendiocarb may offer more protection against malaria infection than deltamethrin on Bioko. This could explain the 2013 island wide rise in malaria prevalence which occurred after spraying was switched to deltamethrin. As local vectors remain fully susceptible to bendiocarb whilst there is evidence of phenotypic and metabolic resistance to pyrethroids, it is likely that deltamethrin is less effective than bendiocarb on Bioko.

## Abbreviations

IRS: indoor residual spraying
WHO: World Health Organization
BIMCP: Bioko Island Malaria Control Project
LLIN: long-lasting insecticidal net
RDT: rapid diagnostic test
WHOPES: World Health Organization Pesticide Evaluation Scheme
